# Solitary Extramedullary Plasmacytoma of the Head and Neck: A Report of Three Cases Treated With Curative Radiotherapy and a Review of the Dose-Control Relationship

**DOI:** 10.7759/cureus.38512

**Published:** 2023-05-03

**Authors:** Irving Sanchez, David Oñate, Tania Hernandez, Victor Ruiz, Omar Diaz, Janie S Munoz, Luis H Bayardo, Alejandro Villalvazo, Emanuel Gamez, Enrique Gutierrez-Valencia

**Affiliations:** 1 Radiation Oncology, Western National Medical Center, Mexican Institute of Social Security (IMSS) University Center for Health Science (CUCS) University of Guadalajara, Guadalajara, MEX; 2 School of Medicine, Universidad Autónoma de Guadalajara, Zapopan, MEX; 3 Pathology, Western National Medical Center, Mexican Institute of Social Security (IMSS) University Center for Health Science (CUCS) University of Guadalajara, Guadalajara, MEX; 4 Radiation Oncology, Princess Margaret Cancer Centre, University of Toronto, Toronto, CAN

**Keywords:** oropharyngeal neoplasms, nasopharyngeal neoplasm, multiple myeloma treatment, plasma cell tumor, radical radiotherapy, 3-d radiotherapy, head and neck neoplasms, laryngeal neoplasms, larynx preservation, solitary extramedullary plasmacytoma (sep)

## Abstract

Solitary plasmacytoma is an uncommon hematologic malignancy characterized by the monoclonal proliferation of abnormal plasma cells in the bone or extramedullary tissues and the absence of other multiple myeloma-defining clinical characteristics. Mostly, solitary extramedullary plasmacytoma (SEP) occurs in the head and neck region, also called solitary extramedullary plasmacytoma of the head and neck (SEPHN). Although the standard of care for SEPHN is not well established, either a surgical approach or localized external beam radiotherapy (EBRT) can be used as a definitive treatment. Due to the high radiosensitivity of SEPHN, EBRT has been associated with adequate therapeutic effects in the management of SEP, with the advantage of being a noninvasive modality that yields high rates of local control with a reasonable toxicity profile. We present a case series of three patients with SEPHN treated with EBRT at our institution with clinical outcomes.

## Introduction

Solitary plasmacytoma is part of the spectrum of plasma cell neoplasms. It presents as a single mass of monoclonal plasma cells located in bone (medullary), or soft tissues (extramedullary), not in contact with the bone [1], in the absence of multiple myeloma-defining criteria, for example, additional bone lesions, end-organ damage, plasmacytosis in bone marrow, anemia, and hypercalcemia [2].

Solitary extramedullary plasmacytoma (SEP) represents 3% of all plasma cell neoplasms and <1% of head and neck tumors [3]. Most SEP occurs in men with a 3:1 ratio and a peak incidence between 50 and 70 years of age [4].

Eighty percent of SEP appears in the head and neck region [5]. The most common sites of SEP of the head and neck (SEPHN) are the nasal cavity, paranasal sinuses, and nasopharynx, with an extremely rare incidence in other sites such as the larynx, which only represents 6% to 18% of all SEPHN [6]. Clinical presentation varies according to the site involved, including mass effect, epistaxis, upper airway obstruction, dysphonia, dysphagia, headache, and motor cranial nerve impairment [4]. Definitive external beam radiotherapy (EBRT) is the treatment of choice, with doses ranging from 30 to 60 Gy [7-9]. Patients diagnosed with SEPHN require close follow-up by hematologists as they show a rate of progression to multiple myeloma of 25% to 35% at 10 years [1], with a higher risk in those with M-protein persistence [10].

## Case presentation

Case #1

A 58-year-old male with 25-pack-year smoking and without any family history of cancer and a singer presented to an otolaryngology clinic with a five-month history of hoarseness. He denied any additional head and neck symptoms.

An office evaluation with laryngoscopy revealed a 2 cm ulcerated lesion in the left true vocal cord with normal mobility. A biopsy from this lesion revealed an ulcerated plasmacytoma comprising kappa-positive and lambda-negative cells. A diagnostic workup for multiple myeloma with bone marrow biopsy, Bence-Jones proteins in urine, bone imaging studies, and serum electrolytes ruled out multiple myeloma and confirmed the diagnosis of solitary plasmacytoma of the larynx.

**Figure 1:**
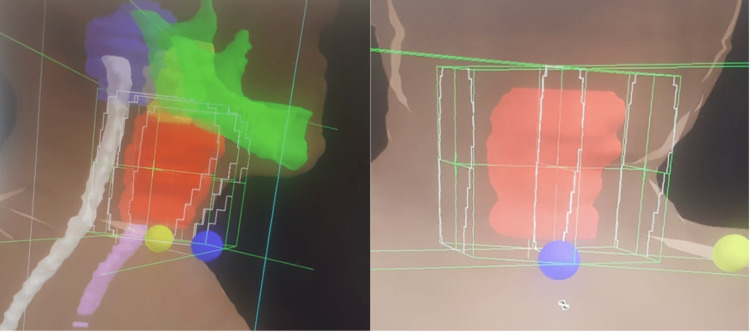
Radiation treatment plan illustrating 3DCRT beam arrangement for laryngeal plasmacytoma; red, PTV; green, mandible; white, spinal cord; pink, esophagus. 3DCRT, three-dimensional conformal radiation therapy; PTV, planning target volume

The patient was referred to the radiation oncology department and treated with EBRT to a dose of 40 Gy in 20 fractions over four weeks, using a three-dimensional conformal radiation therapy (3DCRT) technique, with opposed lateral portals (Figure 1). The clinical target volume (CTV) included the whole larynx plus a 0.5 cm expansion to make the planning target volume (PTV). During treatment, the patient had grade 1 dysphagia, with no other significant toxicities. Complete clinical response to EBRT was noted at a four-month follow-up on laryngoscopic evaluation.

At 12- and 24-month follow-up visits, laryngoscopy was performed, maintaining a complete clinical response, and subglottic stenosis of approximately 50% was reported.

Case #2

A 50-year-old female with a family history of her mother with breast cancer and a sibling with unspecified leukemia presented in March 2020 with a six-month history of odynophagia and dysphagia to solids.

**Figure 2:**
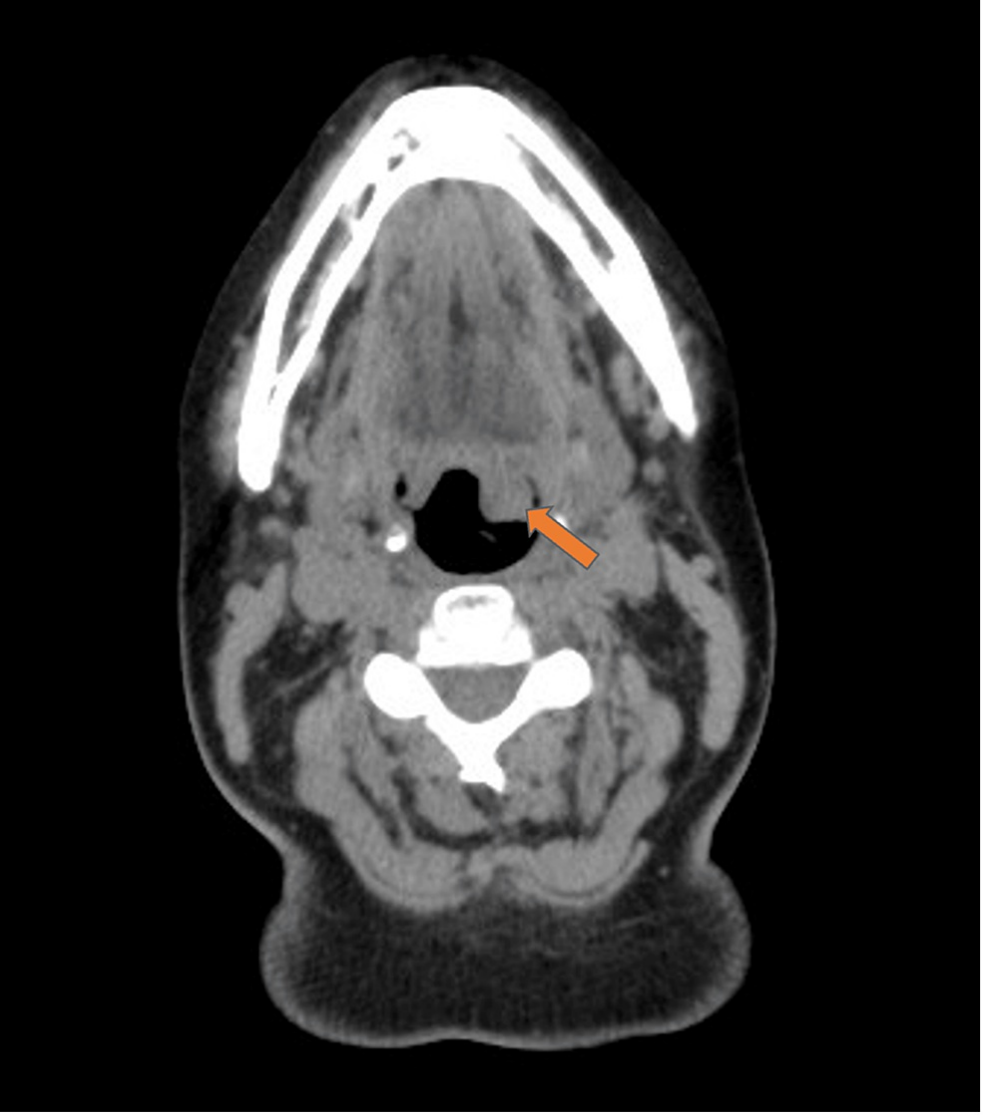
Computed tomography showing the base of the tongue nodular lesion (arrow).

A computed tomography scan showed several solid lesions in the left base of the tongue extending to the floor of the mouth, and the largest lesion measured 19 mm × 16 mm × 20 mm. No enlarged cervical lymph nodes were found (Figure 2). A biopsy of the most extensive lesion was taken, demonstrating subepithelial infiltration by plasma cells arranged in nests CD138+ and CD56+. The patient underwent a bone marrow biopsy that showed normal cellularity for her age. Immunoglobulin (IgG) of 1,474 mg/dL and kappa and lambda serum proteins were found. The diagnosis of SEP of the base of the tongue was established.

**Figure 3:**
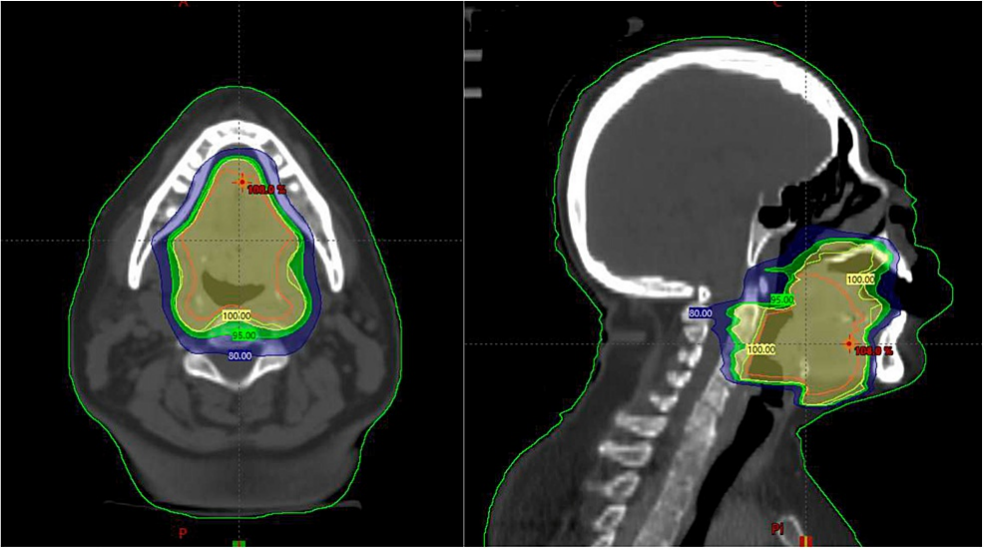
Isodose distribution of the prescribed dose throughout the irradiated treatment volume at the base of the tongue and normal tissue: yellow, 100%; green, 95%; and blue, 80%.

The patient was treated with curative-intent EBRT to a dose of 45 Gy in 25 fractions over five weeks using volumetric modulated arc therapy (VMAT) and image-guided radiation therapy (IGRT) (Figure 3). The gross tumor volume (GTV) included the visible macroscopic disease of the base of the tongue. The CTV included the GTV and a 1 cm expansion and the preepiglottic space, followed by a 0.5 cm expansion to create the PTV. The patient presented grade 2 oral mucositis and grade 1 radiodermatitis during the last two weeks of treatment.

**Figure 4:**
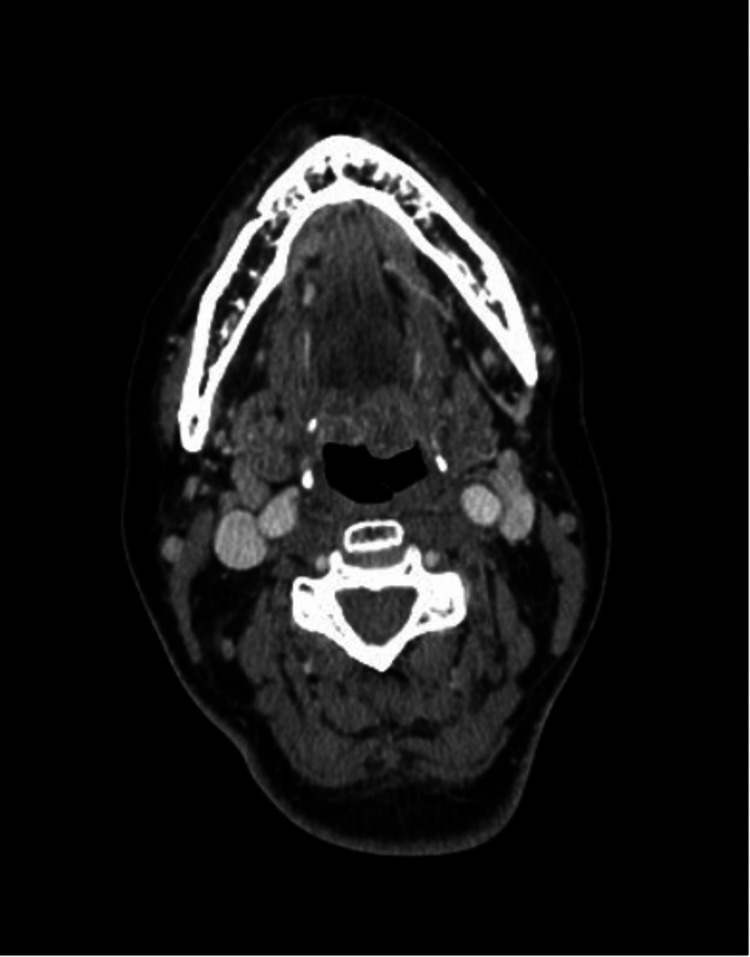
Computed tomography showing no enhancing lesions consistent with complete response.

Grade 1 xerostomia was noted at three months after EBRT without any late side effects. A computed tomography scan at six months revealed no evidence of tumoral activity (Figure 4). A repeated bone marrow biopsy reported 5% of clonal plasma cells without other laboratory abnormalities.

Case #3

**Figure 5:**
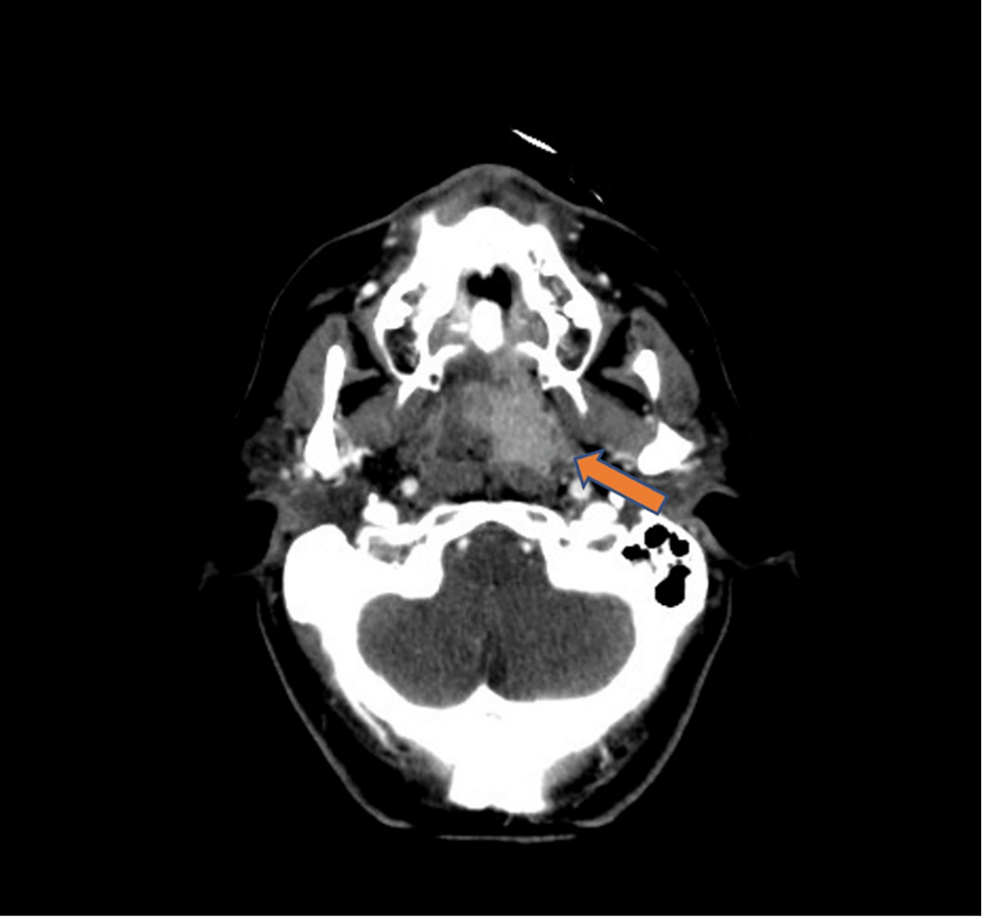
Computed tomography showing a contrast-enhancing mass in the left nasopharynx (arrow).

A 56-year-old male with no relevant medical history presented with repetitive episodes of epistaxis, requiring nasal packing in the emergency department. A nasal endoscopy was conducted in the emergency room, observing profuse active bleeding, impending observing the source of the bleeding, and limiting the procedure to hemostasis only. Computed tomography showed a soft tissue mass in the left nasopharynx occupying the entire space, thereby obstructing both choanae and partially extending into the nasal cavity with homogeneous enhancement upon contrast administration (Figure 5). No cervical lymph nodes are suspicious for metastases were found. A left median meatotomy and ethmoidectomy were performed, including partial resection of the nasopharyngeal tumor.

**Figure 6:**
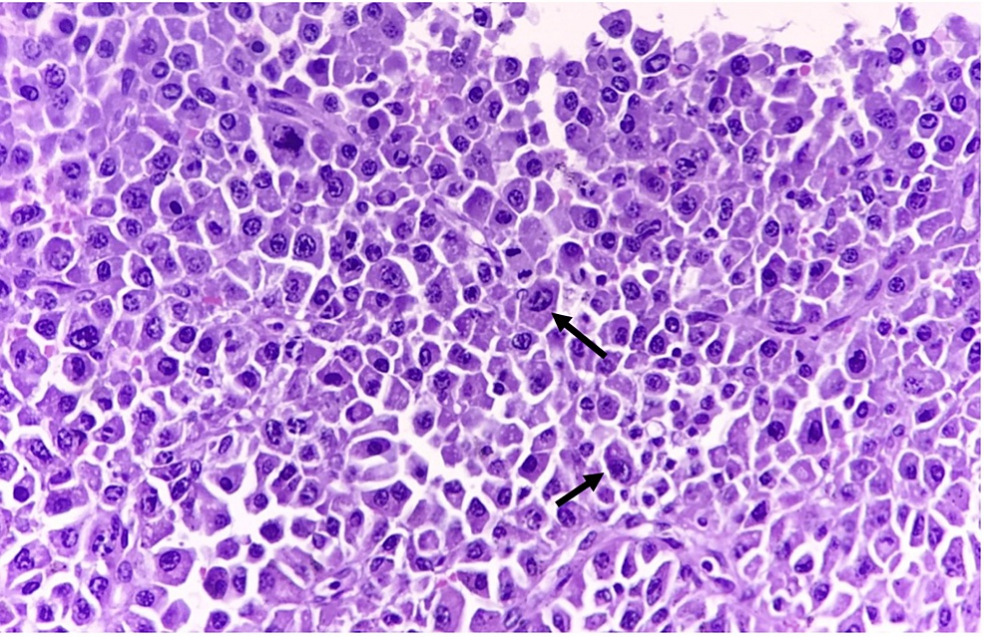
High-power view (40×) revealing the proliferation of atypical plasmacytoid cells with focal pleomorphism. The cells demonstrate medium to large size with a round nucleus, centrally and eccentrically located, with dense cytoplasm and a clear perinuclear zone in the region of the Golgi apparatus (arrows).

The pathology report revealed the presence of cells, with morphological and immunohistochemical findings consistent with plasma cell neoplasia CD138+ and CD56+ in 20% of neoplastic cells (Figure 6).

**Figure 7:**
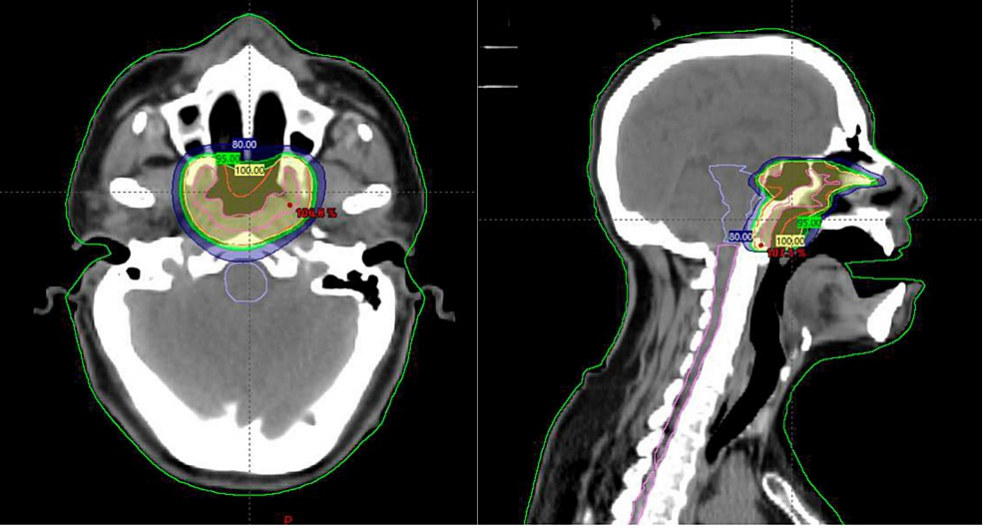
Isodose distributions in color wash representing the dose distribution throughout the treatment volume in the nasopharynx and normal tissue: yellow, 100%; green, 95%; and blue, 80%.

A bone marrow biopsy revealed a normal cellular marrow for age; <1% of clonal plasma cells were observed. A whole-body X-ray bone imaging showed no osteolytic lesions. The patient was evaluated in the radiation oncology department and was treated with EBRT to a dose of 40 Gy in 20 fractions over four weeks with VMAT using IGRT. The CTV included the whole nasopharynx plus a 0.5 cm expansion to create the PTV (Figure 7).

**Figure 8:**
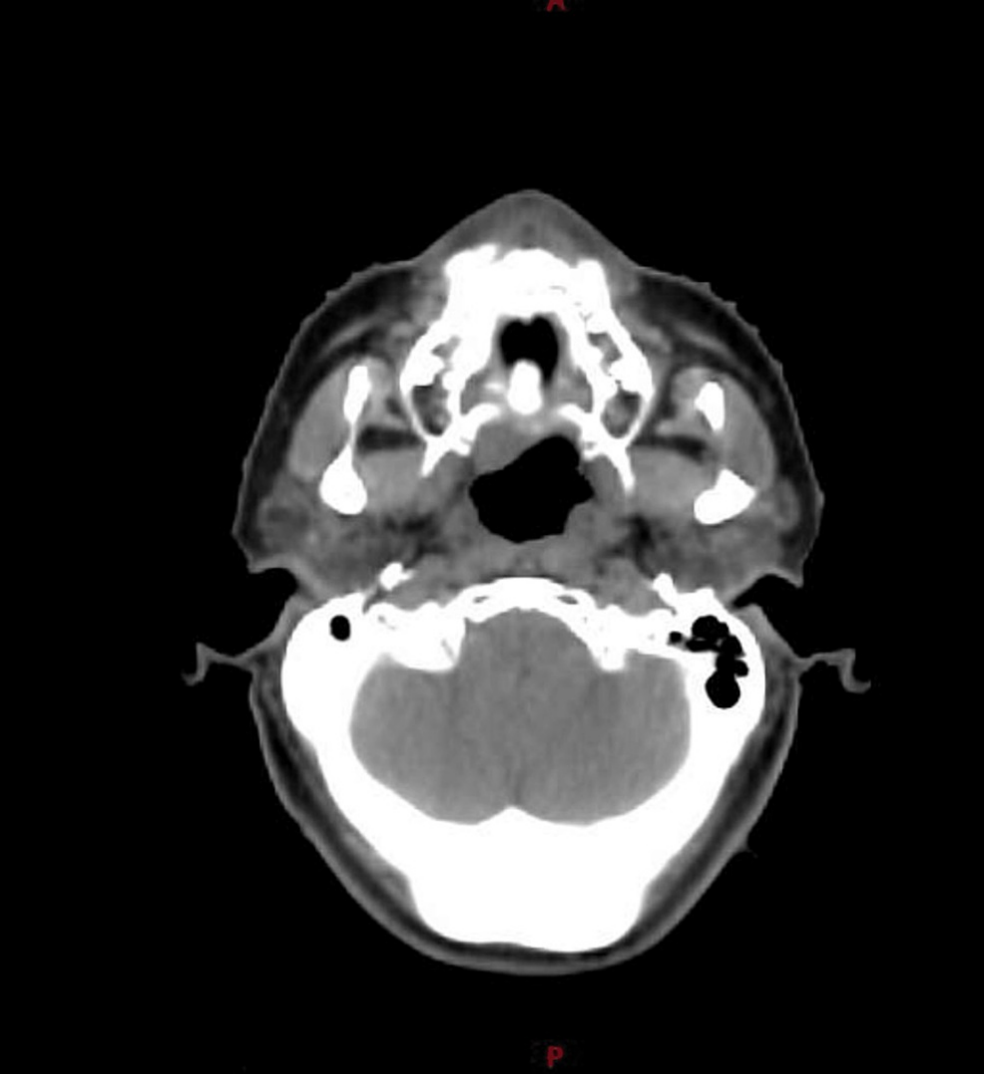
Computed tomography showing no abnormal enhancing lesions in the nasopharynx.

The patient experienced grade 1 radiodermatitis during the treatment with no other toxicities. On a follow-up visit at four months, the head and neck tomography showed no evidence of lesions suggestive of tumoral activity (Figure 8).

## Discussion

Solitary plasmacytoma is part of the spectrum of plasma cell neoplasms. It is further divided into bone and extramedullary plasmacytomas in the absence of multiple myeloma-defining clinical characteristics. SEP comprises 3% of all plasma cell neoplasms and <1% of head and neck tumors [2,3]. Risk factors require to be better established for these entities but have been associated with viral infections such as the Epstein-Barr virus, radiation exposure, chronic stimulation, and gene disorders in the reticuloendothelial system [11].

Currently, a combined therapeutic approach for SEPHN, including surgery, EBRT, and systemic treatments, is usually accepted with no clearly defined standard of care. EBRT is generally proposed as the treatment of choice for SEPHN, thus providing a noninvasive modality compared to surgery and its implications. EBRT is especially useful in SEPHN localized in anatomical sites where the tumor is not easily accessible, making it technically challenging to obtain a complete tumor excision and maintain aesthetic results and functional outcomes such as in tumors affecting the oropharynx and nasopharynx (cases #2 and #3) or when an organ-sparing treatment is desirable as in tumors originating in the larynx (case #1).

Given the high radiosensitivity of SEPHN, radical surgery should be avoided as primary treatment as it can be mutilating [1,2], thereby reserving surgical debulking only for those tumors causing mass effects or neurologic compromises, and is often followed by EBRT or as a sole modality when EBRT contraindications exist [12].

EBRT dose for SEPHN is not well-established, with doses ranging from 30 to 60 Gy in 1.8 to 2 Gy per fraction [7-9]. EBRT alone has been reported as an acceptable and curative modality, achieving control rates of up to 95% at five years in the two largest series of patients reported to date [9,13].

Dose/fractionations recommendations are based on the tumor size, with 40 Gy being prescribed for tumors smaller than 5 cm and 50 Gy for tumors larger than 5 cm [14]. Nonetheless, some studies have shown no dose-response relationship beyond 30 to 35 Gy [8], while others have reported better local control rates with a dose of >40 Gy [15,16]. Moreover, additional evidence from 1997 to 2012 suggests that SEPHN is optimally controlled with a dose between 40 and 50 Gy or higher (Table 1). An important characteristic of these studies was the treatment techniques used before the IMRT and VMAT era.

The EBRT treatment volume comprises the GTV, which includes the observed lesion in computed tomography simulation with or without magnetic resonance imaging. The CTV consists of the GTV plus a 0.5 to 1 cm expansion respecting anatomic boundaries. A PTV to account for daily setup uncertainties during treatment is subsequently created by adding a 0.5 to 1 cm margin [6,17,18]. With new imaging techniques, elective nodal irradiation is not recommended due to the low rate of nodal relapse in the absence of initial nodal involvement [6,17].

**Table 1:** Comparison of SEPHN studies comparing prescribed doses and disease control. EBRT, external beam radiotherapy; 3DCRT, three-dimensional conformal radiation therapy; NR, not reported; 2DRT, two-dimensional radiation therapy

Author (Year)	Number of patients	EBRT technique	Mean prescribed dose (range) (Gy)	Local control at five years (%)	Overall survival at five years (%)
Susnerwala et al. (1997) [6]	25	NR	38.9 (25-50)	88	59.8
Sasaki et al. (2012) [9]	67	3DCRT	50 (30-60)	95	73
Bachar et al. (2008) [13]	68	NR	35 (10-50)	91	76
Liebross et al. (1999) [16]	22	2DRT	50 (40-60)	95	78

Regarding EBRT techniques, the treatment volume and critical structures may largely dictate the choice of treatment modality for each case. Conformal EBRT with parallel fields is commonly used. However, as previously stated, earlier studies were published before IMRT/VMAT techniques were widely implemented worldwide. Hence, in some situations, more conformal techniques such as IMRT and VMAT may be the preferred option to spare critical structures, minimizing toxicity while maintaining therapeutic dosage, usually at the cost of a larger total volume of normal tissue irradiated, but to a lower dose. IGRT delivery may offer a clinically appropriate advantage, particularly for treatment sites that are adjacent to critical dose-limiting normal structures [2,17].

In the present case series, we report the clinical presentation and characteristics of radiotherapy, toxicity, and clinical outcomes of three patients with SEPHN. In case #1, the patient remains disease-free at two-ya ear follow-up but with long-term toxicity presented as subglottic stenosis. Although cases #2 and #3 require longer follow-ups, we believe that the prescribed treatment will provide adequate local control of the disease, as has been described in larger case series [6,9,13,16].

Similarly, we believe that the use of a more modern EBRT technique such as VMAT as in cases #2 and #3 can help reduce long-term side effects, unlike case #1, who was treated at our institution when advanced EBRT techniques were not yet available.

Although patients with SEP show a lower rate of progression to multiple myeloma than patients with solitary bone plasmacytoma (25%-35% vs. 65%-84% at 10 years) [1], prudent follow-up evaluation is recommended, especially in patients with serum M-protein persistence after completion of EBRT as they have a considerably higher risk of multiple myeloma progression than those whose serum M-protein has resolved [10]. The recommended visit intervals are every three to six months, including complete blood count, serum chemistry, immunoglobulins, and bone marrow aspirate and biopsy as indicated. Response assessment using the same imaging studies as for diagnosis is best performed three to six months after completion of treatment and then annually [17].

## Conclusions

SEPHN is an uncommon hematologic malignancy in which the standard of care is not well defined. Nonetheless, it is crucial to understand its high radiosensitivity and biological behavior as, in carefully selected cases, EBRT (3DCRT and IMRT/VMAT) has been associated with excellent control rates. As presented in our case series, EBRT is a well-tolerated treatment with an acceptable toxicity profile providing adequate control of the disease with the dose ranging from 40 to 50 Gy in conventional fractionation.

Moreover, normal tissue sparing and fair aesthetic and functional outcomes are paramount, especially in head and neck subsites. There a surgical resection could be mutilating and can compromise organ functioning as in SEPHN originating in the larynx and oropharynx (cases #1 and #2) or anatomical sites where the tumor is not easily accessible for surgeons or as in tumors affecting the nasopharynx (case #3), making EBRT the treatment of choice.
